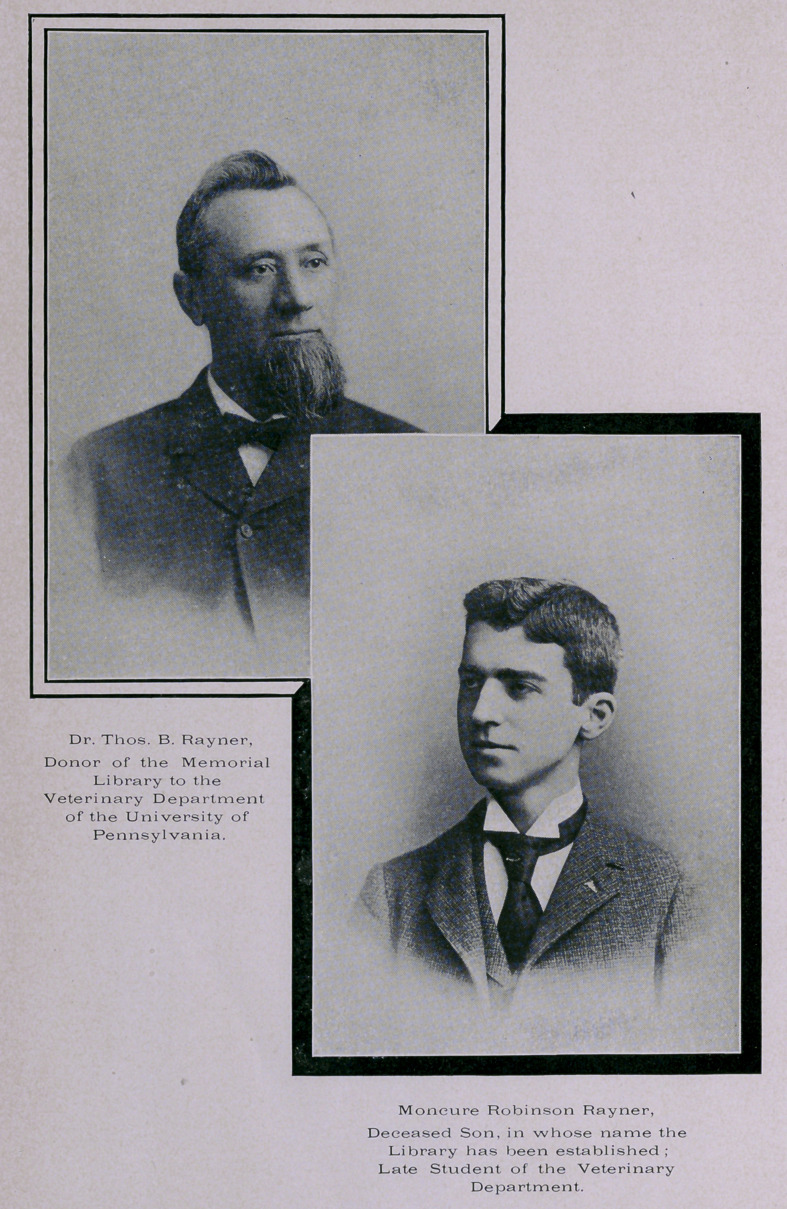# Our Frontispiece

**Published:** 1902-07

**Authors:** 


					﻿EDITORIALS.
OUR FRONTISPIECE.
We are very glad to be able to reproduce in this number of
the Journal the picture of Dr. Thomas B. Rayner and his late
son, Moncure Robinson Rayner, the donor of the new library
to the Veterinary Department of the University of Pennsyl-
vania and the one in whose name it is dedicated. Surely a
grand memorial to one whose life work was planned for the
field of veterinary science, but cut down suddenly, in his
studentship and just on the eve of the age of manhood. Such
gifts attest a parent’s love, bless the donor thereof, and add a
priceless blessing to the future profession.
Minneapolis will prove an attractive city for our convention.
There is much to be seen within the city that is attractive, while
around and about her are beautiful lakes where a day’s stay will be a
pleasant and profitable diversion.
				

## Figures and Tables

**Figure f1:**